# 6-Paradol Alleviates Testosterone-Induced Benign Prostatic Hyperplasia in Rats by Inhibiting AKT/mTOR Axis

**DOI:** 10.3390/plants11192602

**Published:** 2022-10-03

**Authors:** Lenah S. Binmahfouz, Haifa Almukadi, Abdulmohsin J. Alamoudi, Ali M. El-Halawany, Hossam M. Abdallah, Mardi M. Algandaby, Gamal A. Mohamed, Sabrin R. M. Ibrahim, Faraj A. Alghamdi, Majed Al-Shaeri, Ashraf B. Abdel-Naim

**Affiliations:** 1 Department of Pharmacology and Toxicology, Faculty of Pharmacy, King Abdulaziz University, Jeddah 21589, Saudi Arabia; 2 Department of Pharmacognosy, Faculty of Pharmacy, Cairo University, Cairo 11562, Egypt; 3 Department of Natural Products and Alternative Medicine, Faculty of Pharmacy, King Abdulaziz University, Jeddah 21589, Saudi Arabia; 4 Department of Biological Sciences, Faculty of Science, King Abdulaziz University, Jeddah 21589, Saudi Arabia; 5 Preparatory Year Program, Department of Chemistry, Batterjee Medical College, Jeddah 21442, Saudi Arabia; 6 Department of Pharmacognosy, Faculty of Pharmacy, Assiut University, Assiut 71526, Egypt

**Keywords:** benign prostatic hyperplasia, 6-Paradol, AKT, mTOR

## Abstract

Introduction: Benign prostatic hyperplasia (BPH) is a common disease among elderly men. Its pharmacological treatment is still unsatisfactory. 6-Paradol (6-PD) is an active metabolite found in many members of the *Zingiberaceae* family. It was reported to possess anti-proliferative, antioxidant, and anti-inflammatory activities. The present study aimed at exploring the potential of 6-PD to inhibit testosterone-induced BPH in rats as well as the probable underlying mechanism. Methods: Male Wistar rats were divided into 6 groups and treated as follows: Group 1 (control group) received vehicles only, Group 2 testosterone only, Groups 3 and 4 received 6-PD (2.5 and 5.0 mg/kg; respectively) and testosterone, and Group 6 received finasteride and testosterone. Results: Daily treatment of animals with 6-PD at the two dose levels of 2.5 and 5 mg/kg significantly ameliorated a testosterone-induced rise in prostate index and weight. This was confirmed by histological examinations of prostatic tissues that indicated a reduction in the pathological changes as well as inhibition of the rise in glandular epithelial height in 6-PD treated rats. Immunohistochemical investigations showed that 6-PD prevented the up-regulation of cyclin D1 induced by testosterone injections. Further, 6-PD significantly modulated mRNA expression of both Bcl2 and Bax in prostate tissues of testosterone-treated rats in favor of anti-proliferation. It also showed antioxidant activities as evidenced by inhibition of accumulation of malondialdehyde (MDA) and exhaustion of catalase (CAT) activity. In addition, 6-PD displayed significant anti-inflammatory activities as it prevented up-regulation of interleukin-6 (IL-6) and nuclear factor kappa B (NF-κB). Immunoblotting analysis revealed that 6-PD significantly inhibited testosterone-induced activation of AKT and mTOR in prostate tissues. Conclusions: 6-PD protects against testosterone-induced BPH in rats. This can be attributed, at least partly, to its antiproliferative, antioxidant, and anti-inflammatory properties as well as its ability to inhibit activation of the AKT/mTOR axis.

## 1. Introduction

Benign prostatic hyperplasia (BPH) is the noncancerous growth of epithelial cells and smooth muscle within the prostatic transition zone that results in an enlarged prostate [1]. It has been proposed that an enlarged prostate contributes to the overall lower urinary tract symptoms (LUTS) complex in men [2]. Unfortunately, its prevalence increases with age, reaching as high as 50% to 60% for males in their sixties and 80% to 90% for those over 70 [3]. BPH progression is influenced by various risk factors including a sedentary lifestyle, obesity, hypertension, and diabetes [4]. The disease is caused by androgens, specifically dihydrotestosterone (DHT) interacting with the prostatic epithelium and stroma [4]. It develops as a result of an imbalance between cellular proliferation and cell death in favor of epithelial and stromal cell proliferation [5]. In addition, the pathophysiology of BPH comprises oxidative stress, inflammation, sex hormone disturbances, and growth factor imbalance [6]. Drug treatment or management of BPH involves the use of pharmacological agents such as 5-α reductase inhibitors, α-adrenergic blockers, and antimuscarinics [7]. However, they suffer intolerable adverse effects [8,9,10]. This necessitates the development of new pharmacological agents with improved efficacy and safety for the treatment of BPH.

In traditional medicine, many plants in the ginger family, *Zingiberaceae*, are used medicinally. The most well-known herbs are ginger (*Zingiber officinale*), and grain of paradise (*Aframomum melegueta*). These two genera are characterized by the presence of pungent principles such as gingerols, paradol, and shogaols [11]. 6- Paradol also is detected in other *Zingiberaceae* plants such as *Aframomum melegueta* seeds, Curcuma rhizomes, and *Alpinia officinarum* rhizomes [11]. Interestingly, these ingredients have been shown to prevent prostate cancer cell proliferation [12,13] and diabetic complications in the prostate [14]. 6-Shogaol is the primary active constituent of dried ginger, and 6-paradol (6-PD) is synthesized from 6-shogaol via a biotransformation process [15]. Chemically, 6-PD is a vanilloid derivative [1-(4-hydroxy-methoxyphenyl)-3decen-one] [16]. It has a plethora of pharmacological actions including antioxidant [17], anti-inflammatory [18], and anti-bacterial activities [19]. Further, it has been shown to possess neuroprotective [20,21], anti-obesity [22], and anti-ulcerative colitis [23] actions as well as having the potential to alleviate diabetic neuropathy [16]. Further, it has proven anti-tumor and anti-proliferative actions [24,25,26]. 6-PD can inhibit important survival pathways such as AKT and mTOR [27]. The structurally-related compound 6-gingerol has been shown to modulate the alteration in apoptosis-related proteins that has been triggered by testosterone [28]. Therefore, the present study aimed at exploring the potential of 6-PD to inhibit BPH induced by testosterone in rats and to explore the possible fundamental mechanisms.

### 1.1. Western Blot

Lysates were made from prostatic tissues first by 10 s sonication repeated 5 times on ice, then by centrifugation for 10 min at 4 °C and 14,000 rpm. The protein concentration of the resulting lysates was quantified using a Protein Assay Kit I (Catalog # 5000006, Bio-Rad, Hercules, CA, USA). Following that, protein lysates (50 µg) were separated by a 10% Tris-Glycine gel and transferred for 2 h using a semidry transfer cell (Bio-Rad) to a PVDF membrane (ab133411, ABCAM, Cambridge, UK). After separation, membranes were blocked with 5% non-fat dry milk in TBST (10 mM Tris (pH 7), 100 mM NaCl, 0.1% Tween 20) for 30 min; then, they were incubated overnight at 4 °C with one of the following primary antibodies: anti-Akt1/2/3 (sc-81434), anti-p-Akt1/2/3 (sc-514032), anti-mTOR (sc-517464), and anti-p-mTOR antibody (sc-293133) (Santa Cruz Biotechnology Dallas, TX, USA). Membranes were washed thoroughly with TBST and then incubated with a rabbit anti-mouse HRP-conjugated secondary antibody for 1 hr at room temperature at 1/10,000 (Catalog # ab6728) (ABCAM, Cambridge, UK). To detect immunoreactivity, an Enhanced Chemiluminescence kit (GE Healthcare, Piscataway, NJ, USA) was used. β-actin was used as a housekeeping gene using an anti-β-actin antibody (Catalog # ab8226) (ABCAM, Cambridge, UK) and a rabbit anti-mouse HRP-conjugated secondary antibody. Protein expression levels were normalized to β-actin. 

### 1.2. Statistical Analysis

All results are presented as mean ± standard deviations (S.D) and screened for normal distribution using the Shapiro–Wilk’s test. The data were analyzed using one-way analysis of variance (ANOVA), followed by Tukey’s post hoc test in GraphPad Prism version 8 (San Diego, CA, USA). A *p* < 0.05 value was considered significant.

## 2. Results

### 2.1. Prostate Weights and Indices

Initially, the protective effects of 6-PD against BPH induced by testosterone were investigated in terms of changes in prostate weight and index. In this regard, testosterone significantly induced prostate weight and index by 166% and 141%, respectively, relative to control values (Table 1). Yet, co-treatment with 6-PD resulted in significant amelioration of testosterone-induced changes in prostate weight and index by 36% and 31% at 2.5 mg/kg, and by 47% and 42% at 5.0 mg/kg, respectively. These beneficial effects were also associated with finasteride co-treatment in comparison to the group that received testosterone alone.

### 2.2. Histopathological Examination

**Figure 1:**
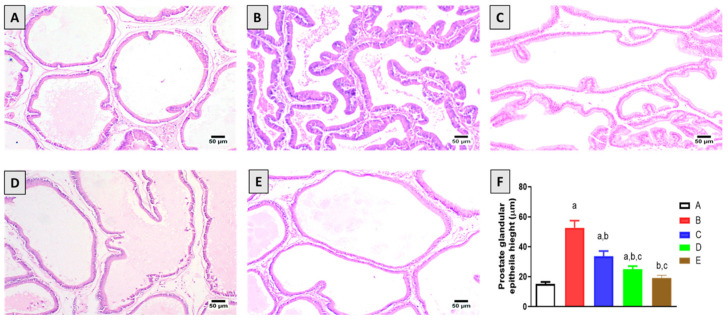
Effect of 6-PD on testosterone-induced pathological changes on prostate tissues: (**A**) control group; (**B**) testosterone (T)-treated group (3 mg/kg) showing hyperplasia of prostatic acini; (**C**) 6-PD (2.5 mg/kg) + T and (**D**) 6-PD (5.0 mg/kg) + T group showing moderate and high restoration of almost normal prostate histology, respectively; and (**E**) finasteride + T group. (**F**) Graphic presentation of prostate glandular epithelial height in the different treatment groups. Data are shown as Mean ± S.D (*n* = 6). a, b, or c: statistically significant from corresponding control, testosterone or T + 6-PD 2.5 mg/kg group, respectively, at *p* < 0.05 using one-way ANOVA followed by Tukey’s post hoc test.

Microscopic examination of the prostate glands of the control group animals revealed normal histology of prostatic acini lined by simple cuboidal epithelium and forming a few inward folds (Figure 1A). Examination of the prostate section obtained from the testosterone-alone group exhibited hyperplasia characterized by proliferation of the epithelial lining into several layers with the folding of the basement membrane forming inward finger-like projections toward the acinar lumen (Figure 1B). Prostate tissues of testosterone-challenged animals and co-treated with 2.5 mg/kg 6-PD showed mild hyperplastic changes in prostate glands with occasional folding with almost normal acini (Figure 1C). Prostatic hyperplasia was alleviated in animals treated with 5.0 mg/kg 6-PD (Figure 1D); some of the examined sections showed mild hyperplastic acini with the folding of the basement membrane into the lumen; meanwhile, some other sections were normal as represented by normal acini with simple cuboidal lining. Marked improvement was observed in testosterone-injected rats co-treated with finasteride (0.5 mg/kg) (Figure 1E), and most of the examined sections showed normal prostatic acini, exhibiting occasional folding. Only a few sections showed mild hyperplasia represented by small inward finger-like projections extending into the acinar lumen. Measurements of the prostate glandular epithelial height indicated significant inhibitory effects of 6-PD with the high dose (5 mg/kg), showing the protective effects comparable to those recorded in the finasteride group (Figure 1E). The prostate glandular epithelial height shows the significant protective effects of 6-PD against testosterone-induced hyperplastic effects (Figure 1F).

### 2.3. Assessment of Cyclin-D1 Expression

**Figure 2:**
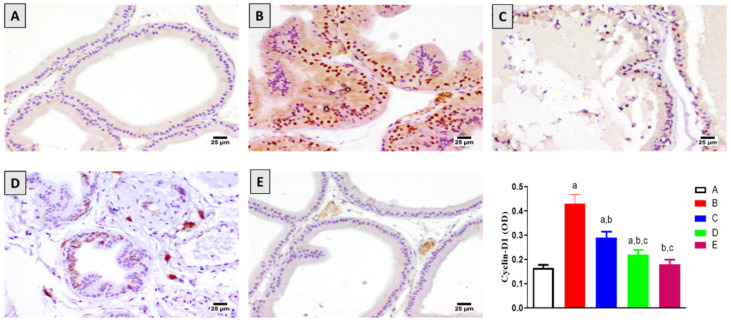
Effect of 6-paradol co-treatment on cyclin-D1 content in the prostatic tissue: (**A**) control group; (**B**) testosterone (T)-treated group (3 mg/kg), (**C**) 6-PD (2.5 mg/kg) + T, (**D**) 6-PD (5.0 mg/kg) + T group and (**E**) finasteride + T group. Data are shown as Mean ± S.D (*n* = 6). a, b, or c: statistically significant from corresponding control, testosterone or T + 6-PD 2.5 mg/kg group, respectively, at *p* < 0.05 using one-way ANOVA followed by Tukey’s post hoc test.

To investigate the anti-proliferative activity of 6-PD, the immunohistochemical expression of the proliferation marker cyclin-D1 was determined. As shown in Figure 2, the number of stained cells was significantly elevated with testosterone administration compared to the control group. However, this increase in cyclin-D1 expression was significantly ameliorated by 6-PD at both tested doses. Co-treatment with 6-PD at 2.5 mg/kg and 5.0 mg/kg resulted in a 33% and 49% decrease in the quantity of stained cells compared to the testosterone-alone group, respectively. The testosterone-induced increase in the number of cyclin-D1 positive cells was also significantly reduced with finasteride administration.

### 2.4. Assessment of Bax and Bcl2 mRNA Expression

**Figure 3:**
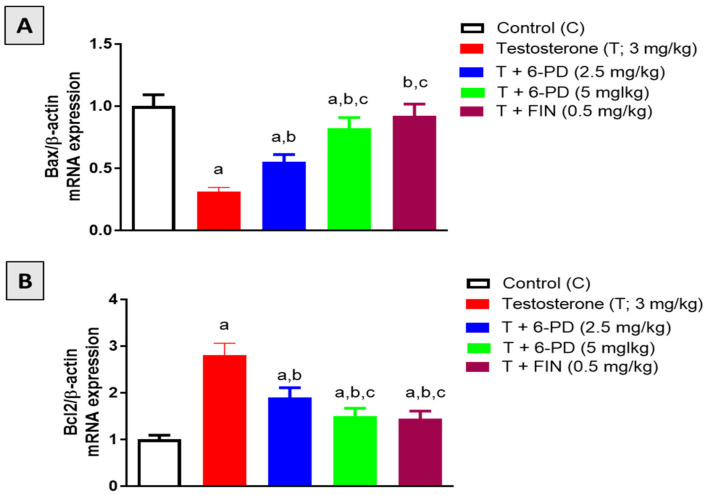
Effect of 6-paradol co-treatment on the mRNA expression of Bax (**A**) and Bcl-2 (**B**) in prostatic tissue. Data are shown as Mean ± S.D (*n* = 6). a, b, or c: statistically significant from corresponding control, testosterone or T + 6-PD 2.5 mg/kg group, respectively, at *p* < 0.05 using one-way ANOVA followed by Tukey’s post hoc test.

The mRNA expression of the pro-apoptotic and anti-apoptotic factors Bax and Bcl2 was investigated to assess the effects of 6-PD on prostatic apoptosis in BPH induced by testosterone. With regards to Bax, testosterone significantly reduced its expression by 69% relative to the control value, whereas this decrease was significantly attenuated by 6-PD by 77% and 164% at 2.5 mg/kg and 5.0 mg/kg, respectively (Figure 3A). On the other hand, Bcl2 expression was significantly induced by testosterone administration, and this increase was significantly ameliorated by 33% and 47% upon co-treatment with 6-paradol at 2.5 mg/kg and 5 mg/kg, respectively (Figure 3B). Comparable variations in the expression of Bax and Bcl2 mRNAs were also observed when testosterone was administered with finasteride.

### 2.5. Assessment of Oxidative Stress Markers

**Figure 4:**
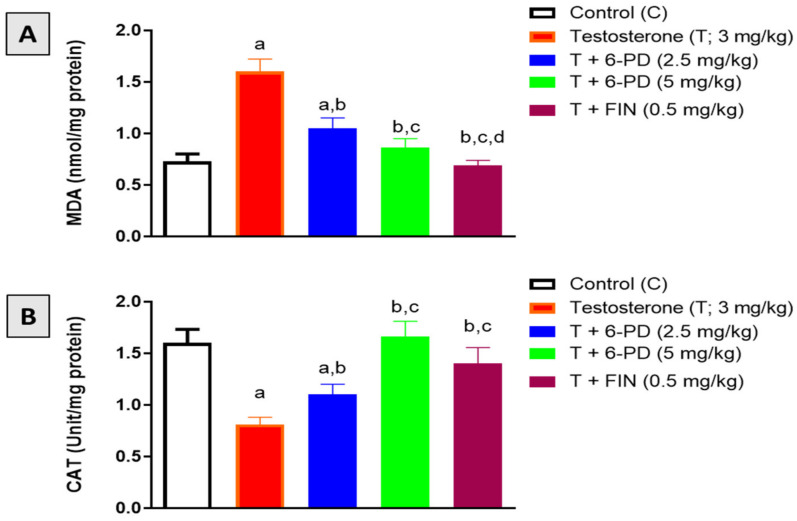
Effect of 6-paradol co-treatment on MDA content (**A**) and CAT activity (**B**) in prostatic tissue. Data are shown as Mean ± S.D (*n* = 6). a, b, c or d: statistically significant from corresponding control, testosterone, T + 6-PD 2.5 mg/kg group or T + 6-PD 5.0 mg/kg group respectively, at *p* < 0.05 using one-way ANOVA followed by Tukey’s post hoc test.

MDA content and CAT enzymatic activity were assessed in this study to investigate the antioxidative potential of 6-PD in prostatic tissues. Testosterone exposure resulted in increased content of the lipid peroxidation marker MDA, whereas 6-PD at 2.5 and 5.0 mg/kg significantly inhibited this increase in MDA by 35% and 46%, respectively (Figure 4A). On the other hand, CAT enzymatic activity was reduced in prostatic tissues of testosterone-treated rats. Yet this testosterone-induced exhaustion of CAT activity was almost normalized by the 6-PD treatment (Figure 4B). Testosterone-induced changes in oxidative stress markers in rat prostates were also prevented by finasteride co-administration.

### 2.6. Assessment of Inflammation Markers

**Figure 5:**
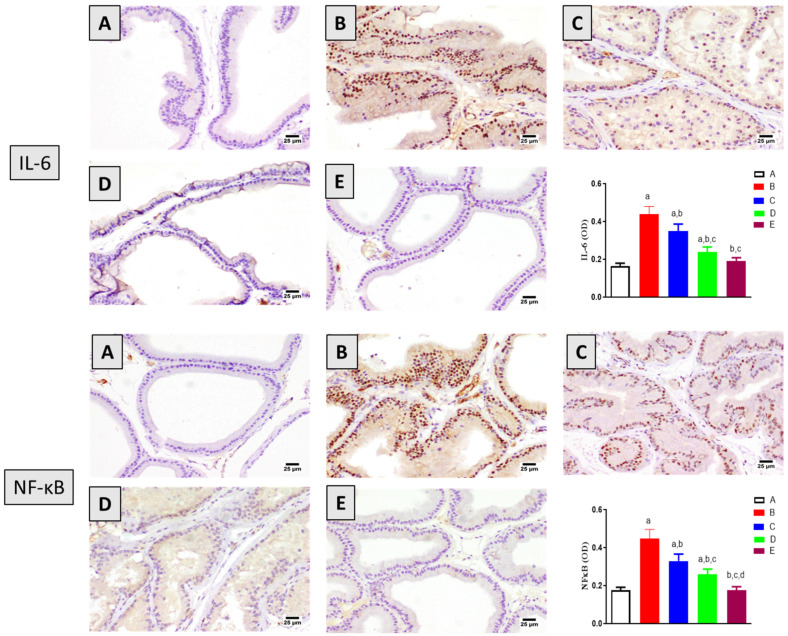
Effect of 6-paradol co-treatment on IL-6 and NF-кB content in the prostatic tissue: (**A**) control group; (**B**) testosterone (T)-treated group (3 mg/kg), (**C**) 6-PD (2.5 mg/kg) + T, (**D**) 6-PD (5.0 mg/kg) + T group and (**E**) finasteride + T group. Graphic presentation of optical density quantification. Data are shown as Mean ± S.D (*n* = 6). a, b, c or d: statistically significant from corresponding control, testosterone, T + 6-PD 2.5 mg/kg group or T + 6-PD 5.0 mg/kg group respectively, at *p* < 0.05 using one-way ANOVA followed by Tukey’s post hoc test.

The anti-inflammatory potential of 6-PD was also investigated in the context of testosterone-induced BPH. As shown in Figure 5, testosterone significantly increased the expression of the inflammatory markers IL-6 and NF-кB in rat prostates. However, 6-PD significantly ameliorated these testosterone-induced changes. At 5.0 mg/kg, 6-PD significantly ameliorated the induced expression of IL-6 and NF-кB by around 46% and 43%, respectively. These anti-inflammatory effects were also associated with finasteride treatment in testosterone-induced BPH.

### 2.7. Assessment of p-AKT and p-mTOR Expression

**Figure 6:**
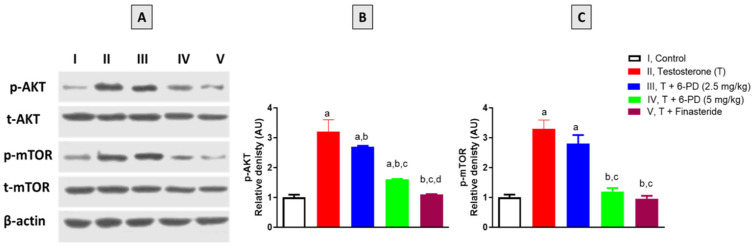
Effect of 6-paradol on the prostatic expression of p-AKT and p-mTOR (**A**) in testosterone-treated rats. Lanes I, II, III, IV, and V represent control, testosterone (T), T + 6-paradol (6-PD) 2.5 mg/kg, T + 6-paradol (6-PD) 5.0 mg/kg, and T + finasteride, respectively. Densitometric data of p-AKT and p-mTOR expression demonstrated in bar charts (**B**,**C**) are mean ± SD (*n* = 6). Data are shown as Mean ± S.D (*n* = 6). a, b, c or d: statistically significant from corresponding control, testosterone, T + 6-PD 2.5 mg/kg group or T + 6-PD 5.0 mg/kg group respectively, at *p* < 0.05 using one-way ANOVA followed by Tukey’s post hoc test.

The final set of experiments was carried out to investigate the effects of 6-PD on the expression status of AKT and mTOR. As shown in Figure 6A,B, phosphorylation of AKT was significantly induced by testosterone exposure in prostatic tissues. Yet, this increase in the content of p-AKT was significantly ameliorated by 6-PD (5.0 mg/kg) by 15% and 100%, respectively. Testosterone exposure also led to increased p-mTOR content in prostatic tissues, whereas 6-PD co-treatment significantly prevented this increase in p-mTOR content at 5.0 mg/kg by 175% (Figure 6A,C). In a similar vein, co-administration of finasteride significantly prevented the testosterone-induced changes in the expression status of AKT and mTOR in rat prostates (Figure 6).

## 3. Discussion

BPH is a common disease among the elderly male population and is associated with irritating side effects that have a negative impact on the quality of life of affected men [29]. It is characterized by the noncancerous enlargement of the prostate gland due to an imbalance between cellular proliferation and cell death [30]. The pathology of the disease is complex. However, oxidative stress and inflammation are important contributors to the initiation and progression of BPH [31]. The use of adrenergic -1 blockers and 5- reductase inhibitors in the treatment of BPH is associated with several intolerable side effects [32,33,34]. 6-PD is a pungent compound found in some members of *Zingiberaceae*, such as ginger (*Zingiber officinale*), and grain of paradise (*Aframomum melegueta*). It possesses a plethora of pharmacological activities including antioxidant, anti-inflammatory, and anti-proliferative actions [17,18,26]. Furthermore, 6-PD was shown to inhibit critical pathways for survival including AKT and mTOR [27]. Hence, this study aimed to investigate the probable effect of 6-PD to inhibit BPH induced by testosterone in rats.

Our data indicated that co-treatment with 6-PD significantly attenuated testosterone-induced increases in prostate weights and indices. These findings were also confirmed by histopathological examination, as 6-PD treatment was associated with significant amelioration of the increase in glandular epithelial height as well as the histopathological signs of prostatic hyperplasia. These observations are in harmony with the anti-proliferative activity of 6-PD reported in several studies [24,25,26]. 6-PD was also associated with chemo-preventive effects against buccal pouch carcinogenesis, which can be attributed to its anti-lipid peroxidative and antioxidant activities [24,35]. Further, it noteworthy to report that 6-PD prevented body weight gain in testosterone-treated animals. This is in harmony with its reported anti-obesity activity [22]. Interestingly, the findings of the current study also highlight the antioxidant potential of 6-PD as it significantly attenuated the exhaustion of CAT activity and accumulation of lipid peroxides in prostate tissues of testosterone-treated rats. As previously reported, oxidative stress plays a role in the pathogenesis of testosterone-induced BPH [36]. Therefore, the protective effects associated with 6-PD in this study could be attributed in part to its antioxidant activity. This gains indirect support from the findings of several studies that correlate the anti-BPH properties of phytochemicals with their antioxidant activities [37,38].

In this study, testosterone administration resulted in a shift in the balance toward cell proliferation in prostatic tissues. This was evidenced by the decreased expression of Bax and increased expression of Bcl2 and cyclin-D1 associated with testosterone administration. This is consistent with other studies describing the effects of testosterone on BPH [39,40]. 6-PD at both tested doses significantly attenuated these pro-proliferative changes in the expression of Bax, Bcl2, and cyclin-D1. These findings are supported by the ability of the structurally related compound 6-gingerol to modulate testosterone-mediated alterations in the expression of Bax and Bcl2 [28]. This is also in line with reports on the inhibitory activity of 6-Shogaol, the precursor of 6- PD, on the expression of cyclin-D1 in prostate and other cell types [41,42].

Inflammation in the prostate is a significant driver of BPH progression as it is strongly correlated with clinical symptom score and prostate volume [43,44]. Several studies on experimental animals demonstrated that inflammation mediates testosterone-induced BPH, whereas agents with anti-inflammatory activities were found to have protective effects against BPH development [39,45]. In this study, it was found that the expression of IL-6 and NF-кB was up-regulated by testosterone administration. Yet, 6-PD significantly ameliorated the increased expression of these inflammatory mediators. These findings are consistent with previous reports highlighting the anti-inflammatory activity of 6- PD [20,23]. Furthermore, the beneficial effects of 6- PD in terms of ameliorating the increase in NF-кB could be stemmed from its antioxidant action because oxidative stress is known to illicit inflammatory reactions via the activation of transcription factors such as NF-кB [46].

It was also shown, in the current study, that 6-PD significantly ameliorated the increased expression of p-AKT associated with testosterone. AKT signaling is known to play an important role in the development of BPH as its expression is directly correlated with prostate size [47,48]. Activation of AKT signaling was also found to disrupt the balance between Bax and Bcl2 in prostate cell lines [49]. These findings are also in line with previous reports demonstrating the potential of 6-PD to suppress cell proliferation and inflammation via inactivating AKT/mTOR signaling pathways [26,27]. This is in harmony with the results of the current study as 6-PD was also found to significantly normalize the expression of p-mTOR in the prostatic tissues of testosterone-treated rats. Increased phosphorylation of AKT and mTOR was found to promote stromal cell proliferation in BPH [50], hence reducing the levels of p-AKT and mTOR could be at least partly responsible for the protective effects of 6-PD observed in this study.

In conclusion, 6-PD exhibits preventive effects against testosterone-induced BPH in rats. This can be attributed, at least in part, to its antiproliferative, antioxidant, and anti-inflammatory properties, as well as its ability to inhibit AKT/mTOR axis activation.

## 4. Materials and Methods

### 4.1. Chemicals

Testosterone enanthate was a kind gift from Chemical Industries Development Co. (CID), Giza, Egypt. Finasteride was purchased from Sigma-Aldrich (St. Louis, MO, USA). All remaining chemicals were of the highest commercial analytical grade. 

### 4.2. Isolation of 6-PD

6-PD was isolated from the methanolic extract of Aframomum melegueta seeds as previously reported [51]. Characterization of the isolated compound was confirmed by ^1^H and ^13^C NMR; meanwhile, its purity was confirmed by HPLC (Figures S1–S3).

### 4.3. Animals

A total of 30 ten-week-old male Wistar rats (150–190 g) were purchased from the Faculty of Pharmacy, King Abdulaziz University, Jeddah. Rats were kept in air conditioning on a half-day light/dark cycle and had unlimited access to water and a standard food pellet diet for one week of acclimatization prior to the study. All procedures were carried out under the National Institute of Health’s guidelines for the care and use of laboratory animals (NIH Publications No. 8023, revised 1978). The Research Ethics Committee at the Faculty of Pharmacy, King Abdulaziz University, Jeddah, Saudi Arabia, approved all animal procedures as ethical (Reference No. PH-1444-02).

### 4.4. Experimental Design and Animal Treatment

6-PD, testosterone enanthate, and finasteride were dissolved in olive oil. Thirty rats were randomly divided into five groups (6/group) and treated five days per week for four weeks. Group 1 (control group) received olive oil (5 mL/kg; PO) and olive oil (1 mL/kg; SC). Group 2 received olive oil (5 mL/kg; PO) and testosterone (3 mg/kg; SC). Groups 3 and 4 received 6-PD (2.5 and 5.0 mg/kg; respectively) and testosterone (3 mg/kg; SC). Group 6 received finasteride (0.5 mg/kg; PO) and testosterone (3 mg/kg; SC). The dosing volume was kept at 5 mL/kg for oral treatments and 1 mL/kg for SC injections. Oral treatments were given 1 h before SC injections.

Rats were weighed three days after the last dose, then anesthetized with xylazine (80 mg/kg, IP) and ketamine (80 mg/kg, IP), followed by cervical dislocation and prostate harvesting. For histological and immunohistochemical examinations, portions of the prostatic ventral lobes were fixed in 10% neutral buffered formalin. Another portion of the prostatic tissues was treated with the RNeasy Mini Kit (Qiagen, Hilden, Germany) for real-time polymerase chain reaction (RT-PCR) whereas the remainder was frozen in liquid nitrogen and stored at −80 °C for biochemical analysis and immunoblotting. 

### 4.5. Histological Examination

The formalin-fixed prostatic tissues were subjected to the paraffin embedding process. Subsequently, sections of the paraffin-embedded tissue (5 μm thickness) were deparaffinized, rehydrated, and stained with hematoxylin and eosin (H&E) for histological examination. At least three stained sections were photographed and then were used to calculate prostate glandular epithelial height by Image J software (1.46 a, NIH, Bethesda, MD, USA).

### 4.6. Assessment of Oxidative Stress Biomarkers 

Malondialdehyde (MDA) content and catalase (CAT) enzymatic activity in prostate homogenates were determined using the following respective commercial kits MD-2528 and CA-2516 (Biodiagnostics, Cairo, Egypt), according to the manufacturer’s instructions. 

### 4.7. Immunohistochemical Staining

Deparaffinized tissue sections were gradually rehydrated using serial dilutions of ethanol before being boiled for 5 min in sodium citrate buffer (10 mM, pH 6.0), and then processed for immunostaining. The tissue sections were blocked for 2 h in 5% bovine serum albumin (BSA) in tris-buffered saline (TBS). Slides were then incubated for 12 h at 4 °C with primary antibodies against: cyclin-D1 (Catalog # ab16663), IL-6 (Catalog # ab9324), and NF-кB p65 (Catalog # ab194726) (ABCAM, Cambridge, UK). Following a TBS rinse, the sections were incubated with the corresponding secondary antibody. A light microscope was used to visualize the slides and a CDD camera to capture the photomicrographs. Optical density (OD) was quantified using Image J software (1.46 a, NIH, Bethesda, MD, USA) from a minimum of three sections per rat.

### 4.8. mRNA Expression of Bax and Bcl-2

The mRNA expression of Bax and Bcl-2 was evaluated using a Real-Time Polymerase Chain Reaction (RT-PCR) assay. TRIzol was used to isolate total RNA from prostatic tissues and its purity and concentration were measured using a spectrophotometer (at A260/A280). First-strand cDNA was synthesized from total RNA using the Omniscript RT kit (Catalog # 205113, Qiagen, MD, USA). Thereafter, RT-PCR reactions were carried out with a SYBR Green Master Mix (Qiagen, MD, USA). The sequences of the primer sets for each gene are shown in Table 2. Primers were ordered from Sigma-Aldrich (Gillingham, UK). The data were normalized f β-actin and analyzed using the delta-delta Ct (ΔΔCt) method calculation [52].

## Figures and Tables

**Table 1:** Effect of 6-PD on prostate weight and prostate index in testosterone-induced BPH in rats.

Parameter	Control	Testosterone (T, 3.0 mg/kg)	T + 6-PD (2.5 mg/kg)	T + 6-PD (5.0 mg/kg)	T + Finasteride (0.5 mg/kg)
Final body weight (g)	265 ± 33.1	291 ± 37.3	271 ± 35.2	262 ± 30.4	256 ± 32.3
Prostate weight (mg)	311 ± 26.6	830 ^a^ ± 55.1	530 ^a,b^ ± 46.2	433 ^a,b,c^ ± 47.1	370 ^b,c^ ± 43.2
Prostate index × 10^3^	1.2 ± 0.14	2.9 ^a^ ± 0.30	1.98 ^b^ ± 0.28	1.67 ^b^ ± 0.18	1.45 ^b^ ± 0.18

Results are shown as Mean ± S.D (*n* = 6). To calculate the prostate index, the prostate weight was divided by the body weight of the rat and multiplied by 10^3^. Results were considered significantly different when at *p* < 0.05 using one-way ANOVA followed by Tukey’s post hoc test. a, b, or c: statistically significant from corresponding control, testosterone or T + 6-PD 2.5 mg/kg group, respectively. T is testosterone, 6-PD is 6-paradol.

**Table 2:** Primers sequences used for the analysis of gene expression.

Target Gene	Primer Sequence	Gene Bank Accession Number
**Bax**	**Forward:** 5’-CCTGAGCTGACCTTGGAGCA-3’ **Reverse:** 5’-GGTGGTTGCCCTTTTCTACT-3’	U32098.1
**Bcl-2**	**Forward:** 5’-TGATAACCGGGAGATCGTGA-3’ **Reverse:** 5’-AAAGCACATCCAATAAAAAGC-3’	NM_016993.1
**β-actin**	**Forward:** 5’-5′TCCGTCGCCGGTCCACACCC-3’ **Reverse:** 5’-TCACCAACTGGGACGATATG-3’	NM_031144.3

## Data Availability

Data are contained in the article.

## Supplementary Materials

The following supporting information can be downloaded at: https://www.mdpi.com/article/10.3390/plants11192602/s1, Figure S1. ^1^HNMR spectra of 6-paradol, Figure S2. ^13^CNMR spectra of 6-paradol, Figure S3. 6-Paradol HPLC chromatogram.
