# Supplementary material for: 6-Paradol Alleviates Testosterone-Induced Benign Prostatic Hyperplasia in Rats by Inhibiting AKT/mTOR Axis
Source: Plants (Basel). 2022 Oct 3;11(19):2602. doi: 10.3390/plants11192602 (PMC9571361; doi:10.3390/plants11192602)
Supplement: Supplementary file 1 [file plants-11-02602-s001.zip › plants-1925261-supplementary.pdf]

Supplementary Materials

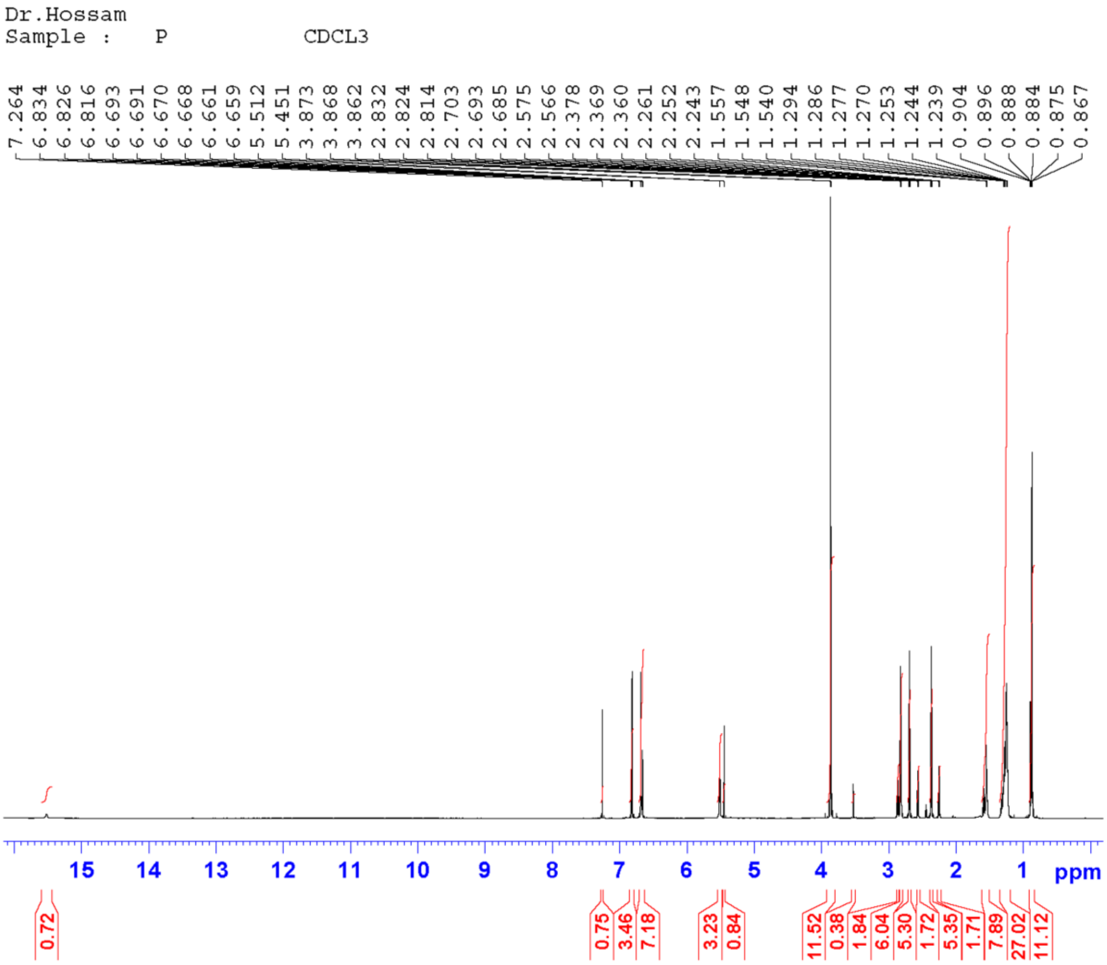

Figure S1. ^{1}HNMR spectra of 6-paradol.

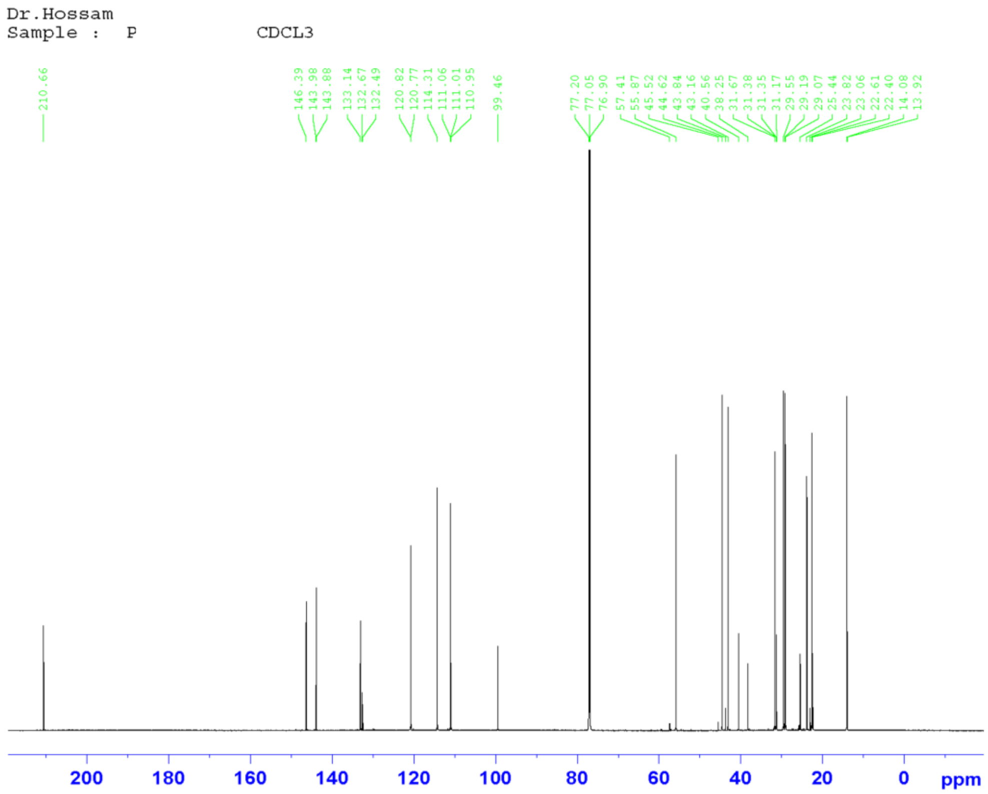

Figure S2. ^{13}CNMR spectra of 6-paradol.

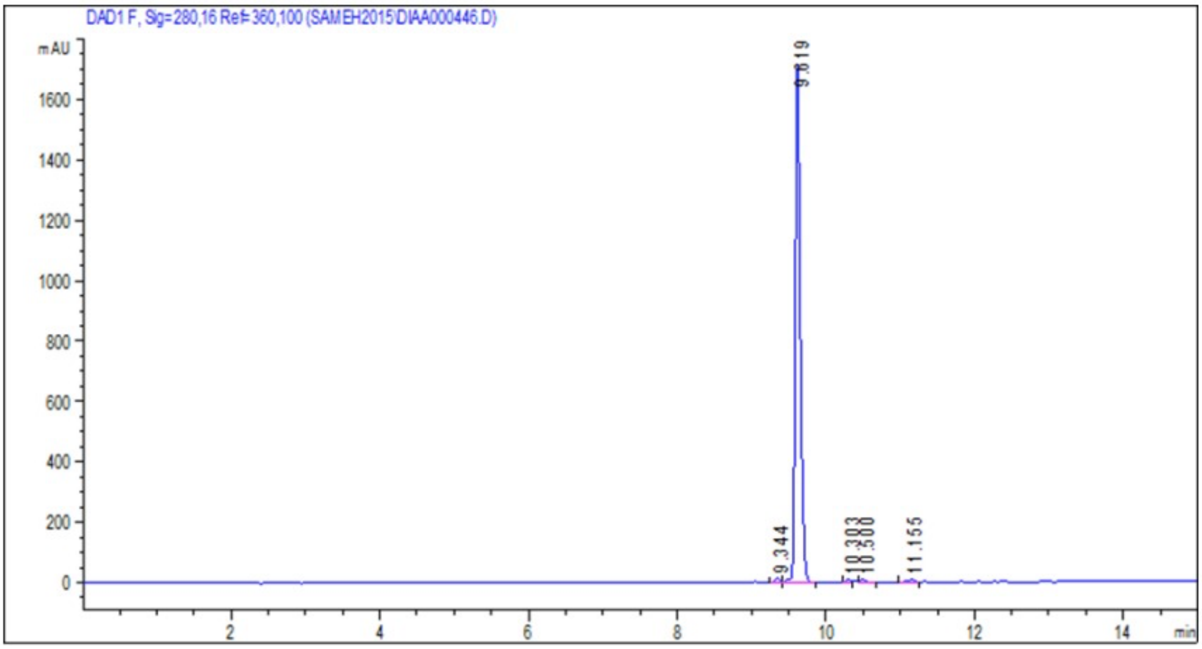

Figure S3. 6-Paradol HPLC chromatogram

HPLC was carried out on Agilent 1200 infinity instrument equipped with automatic injector and DAD detector. Paradol was chromatographed gradiently using a mobile phase A (acetonitrile) and B (0.1% TFA). The gradient elution program was: 35 % A (0-2 min), 35 - 60 % A (2-10 min), 60 - 100 % A (10-12 min) and 100 - 35 % A (12-16 min). The flow rate was 1.0 mL/min and the peaks were monitored 280 nm, respectively. Purity of paradol was 97% at these conditions.
